# Influence of PAS Domain Flanking Regions on Oligomerisation and Redox Signalling By NifL

**DOI:** 10.1371/journal.pone.0046651

**Published:** 2012-10-08

**Authors:** Richard Little, Peter Slavny, Ray Dixon

**Affiliations:** Department of Molecular Microbiology, John Innes Centre, Norwich Research Park, Norwich, United Kingdom; Centre National de la Recherche Scientifique, Aix-Marseille Université, France

## Abstract

Per-ARNT-Sim (PAS) domains constitute a typically dimeric, conserved α/β tertiary fold of approximately 110 amino acids that perform signalling roles in diverse proteins from all kingdoms of life. The amino terminal PAS1 domain of NifL from *Azotobacter vinelandii* accommodates a redox-active FAD group; elevation of cytosolic oxygen concentrations result in FAD oxidation and a concomitant conformational re-arrangement that is relayed via a short downstream linker to a second PAS domain, PAS2. At PAS2, the signal is amplified and passed on to effector domains generating the ‘on’ (inhibitory) state of the protein. Although the crystal structure of oxidised PAS1 reveals regions that contribute to the dimerisation interface, 21 amino acids at the extreme N-terminus of NifL, are unresolved. Furthermore, the structure and function of the linker between the two PAS domains has not been determined. In this study we have investigated the importance to signalling of residues extending beyond the core PAS fold. Our results implicate the N-terminus of PAS1 and the helical linker connecting the two PAS domains in redox signal transduction and demonstrate a role for these flanking regions in controlling the oligomerisation state of PAS1 in solution.

## Introduction

PAS (Per-ARNT-Sim) domains represent highly conserved α/β folds of approximately 110 amino acids that perform diverse signalling functions in proteins from all kingdoms of life [1]. Multiple PAS domains are frequently found in the same protein, often in combination with other signalling domains such as the structurally related GAF domain, underscoring the architectural importance of this module to signal transduction in biological systems [2]. PAS domains integrate the metabolic and energetic status of the surroundings through the binding of small molecules or by sensing environmental stimuli via bound co-factors. Conformational changes resulting from signal sensation initiate signal relay to effector domains within the protein. Alternatively, PAS-mediated signalling can be established through hetero-dimerisation of PAS modules from different proteins or through association between PAS units and alternative protein domains [3].

The architecture of the PAS domain core is defined by a highly conserved five-stranded antiparallel β-sheet around which several α-helices are arranged [4], [5]. Ligand binding PAS domains typically accommodate their co-factor within a spatially conserved cleft formed by the inner surface of the β-sheet and two α-helices termed Eα and Fα. In addition to the core fold, PAS domains commonly possess flanking regions at their N- and C-termini. PAS modules are usually located at the N-terminus of effector proteins and therefore C-terminal extensions serve as linkers to the effector region or to another PAS or other signalling domain in the case of proteins containing multiple signalling modules. Sequences that flank PAS domains are predicted to adopt an α-helical structure and where structures have been determined, the majority adopt an α-helical conformation [2]. Overall, these structures reveal that flanking regions either extend outwards from the PAS domain or pack against the conserved β-sheet of the core structure. However, the native arrangement of these elements may be dependent upon other regions of the protein that may or may not be present in a particular structure.

Multiple sequence alignments of linkers connecting PAS modules with effector domains [2], [6] and linkers between multiple PAS domains [7] reveals the presence of hydrophobic residues displaying a heptad periodicity, characteristic of α-helical coiled-coils. Deletions that remove residues in these amphipathic helical linkers have significant effects on signal transduction, dependent upon their influence on helical rotation within the coiled-coil. This suggests a model whereby signals are transmitted along the coiled-coil linker in the form of torque or helical rotation.

**Figure 1:**
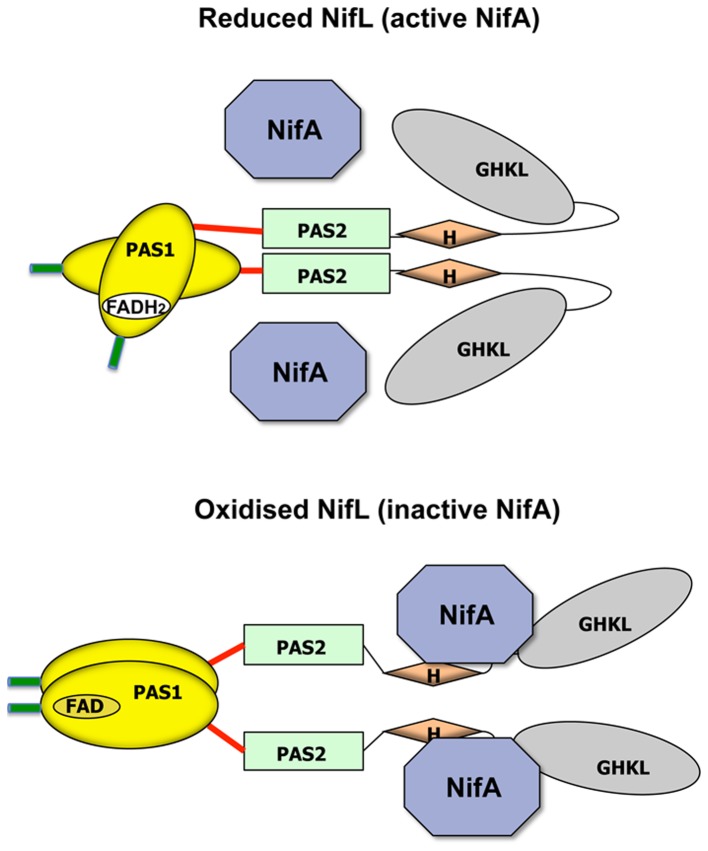
Working model for redox signal transduction by NifL. The cartoon depicts the four structural domains of NifL (PAS1, PAS2, H and GHKL) with NifA shown as a single polygon. Under reducing conditions, NIfL assumes a conformation (“off state") that is not competent to inhibit NifA. When the FAD co-factor in the PAS1 domain is fully protonated, the quaternary structure of PAS1 maintains PAS2 in the dimeric state, resulting in the C-terminal H and GHKL domains assuming a conformation that prevents their interaction with NifA. Under oxidising conditions, NifL assumes the inhibitory “on state" conformation that is competent to form a complex with NifA and consequently inhibit its ability to activate transcription. Oxidation of the FAD moiety results in a quaternary structure change in PAS1, resulting in a movement of the PAS2 protomers that is proposed to trigger rearrangement of the H and GHKL domains. This reorganization promotes access to NifA and the formation of a protein-protein complex that inhibits its activity. The N-terminal flanking region of PAS1 and the linker connecting the PAS1 and PAS2 domains, which are the subject of this work, are shown in green and red respectively.

The NifL protein from the diazotrophic bacterium *Azotobacter vinelandii* is an extensively studied relative of the histidine protein kinases that contains tandem N-terminal PAS domains. NifL functions as a sensor to regulate the expression of nitrogen fixation (*nif*) genes in response to both the oxygen and fixed nitrogen status, by controlling the activity of its partner, NifA, a transcriptional activator belonging to the bacterial enhancer-binding protein family. Under conditions that disfavour nitrogen fixation (excess oxygen or fixed nitrogen) allosteric regulation converts NifL into an “on" state that forms a stoichiometric protein-protein complex with NifA, preventing the latter from activating *nif* transcription [8], [9]. Both PAS domains of NifL are required for redox signal transduction in response to oxygen (Figure 1). The N-terminal PAS domain (PAS1) contains an FAD co-factor that acts as a redox sensitive switch [10], [11], whereas the second PAS module (PAS2) relays the redox signal to the C-terminal H and GHKL domains [12]. In the current model (Figure 1) oxidation of the FAD co-factor in PAS1, initiates a chain of conformational changes that propagate downstream to PAS2, which undergoes quaternary re-arrangement, triggering a proposed repositioning of the C-terminal effector domains [10], [12].

The crystal structure of the oxidised PAS1 domain of NifL reveals a typical PAS fold that dimerises via an interaction between A′α-helices from each subunit and involves residues from the conserved β-sheet [13]. However the first 21 amino acids of NifL (indicated by the green lines in Figure 1) are unresolved in this structure and the properties of the C-terminal linker connecting the two PAS domains (red lines in Figure 1) has not been investigated. Hence questions remain regarding the overall fold of the PAS1 domain in the presence of the flanking regions and the contribution that these elements may make to signal transduction. In this study, we have used a combined genetic and biochemical approach to investigate the role of flanking regions in NifL activity and the association state of the PAS1 domain. Results with deletion variants show that these sequences have a significant influence on oligomerisation of the PAS1 domain and in particular, the length of the C-terminal linker helix is crucial to the generation of signal output and the response to the redox signal.

## Materials and Methods

### Deletion mutagenesis and site-directed mutagenesis of *nifL*

All plasmids used to investigate NifL activity *in vivo* were derived from pPR34, which is a pT7-7 derivative carrying transcriptionally coupled (and independently translated) copies of the *A. vinelandii nifL* and *nifA* genes under the control of a constitutive promoter [14].

Constructs encoding N-terminal truncations of the NifL protein were prepared by amplifying the *nifL* gene from the desired starting codon (using an oligonucleotide that introduces an *Nde*I site upstream of the annealing region) to a region downstream of the *NotI* site in *nifL* using primer MS2Rev (5′-GCGCGAAGAACACGTGGGCCTG-3′). The resulting PCR products were purified (Qiagen PCR purification kit) and digested with restriction endonuclease enzymes, *Nde*I and *Not*I (Roche). The digested fragment was cloned into pPR34 [14].

Site directed mutagenesis to prepare single amino acid substitutions was performed using a two-step PCR approach. One reaction was carried-out with the forward primer T7 (5′- TAATACGACTCACTATAGGG-3′) and a reverse primer carrying the desired mutation. The second reaction was carried-out with the reverse primer NifLrev (5′-GCTCGGGTTGGAGAGCATCAC-3′) and a forward primer carrying the desired mutation. The products from the first step PCR reactions were purified (Qiagen PCR purification kit) and digested with the restriction endonuclease enzymes, *Nde*I (Roche) and either *Mlu*I or *Not*I (New England Biolabs) for cloning into pPR34. Deletions in the linker helix were prepared using a similar two-step PCR procedure with the exception that the forward internal primer contained a 5′ non-annealing tail complementary to the region upstream of the sequence to be deleted.

### ß-galactosidase assays

Growth conditions and β-galactosidase assays to determine NifA transcriptional activity were performed as described previously [15], [16]

### Western blotting

To obtain protein extracts, cultures of *E. coli* ET8000 cells containing the plasmid of interest were grown as for the β-galactosidase assays. To ensure that the cell numbers were equivalent between samples, the volume taken from each culture was adjusted according to differences in OD_600_. The normalised cell samples were centrifuged and the pellet re-suspended in protein loading buffer (125 mM Tris-HCl, 4% sodium dodecyl sulphate, 20% glycerol, 10% β-mercaptoethanol, 0.05% bromophenyl blue, pH 6.8). The proteins were separated by SDS-PAGE and electrotransferred onto nitrocellulose membranes. Membranes were probed with polyclonal antisera against NifL and primary antibodies were detected with alkaline-phosphatase-conjugated anti-rabbit secondary antibodies. Secondary antibodies were detected by staining with 5-bromo-4-chloro-3-indolylphosphate and nitroblue tetrazolium.

### Plasmid construction for protein overexpression and protein purification

All plasmids for protein overexpression were derived from a modified form of the vector pETM11 (EMBL) named pETNdeM-11, in which the *Nco*I site in the multiple cloning region is mutated to yield an *Nde*I site. DNA fragments encoding the required region of NifL were PCR amplified using primers with appropriate restriction sites engineered at their 5′ ends (*Nde*I and *Bam*HI), enabling directional cloning into the expression vector.

In all cases overexpression was carried-out in *E. coli* BL21(DE3)pLysS cells. Cultures were grown aerobically in LB medium and expression from the T7 promoter was induced by the addition of IPTG to a final concentration of 1 mM. Proteins were purified as described previously [10], [12], [17].

### Size-exclusion chromatography

Size-exclusion chromatography was performed over a Superose 12 10/300 Gl column (G E Healthcare) at a flow rate of 0.5 ml min^−1^ in 50 mM Tris-HCl, 10% v/v glycerol, 200 mM NaCl, pH 8.0. Bio-Rad gel filtration standards (thyroglobulin [bovine], γ-globulin [bovine], ovalbumin [chicken], myoglobin [horse] and vitamin B12) were used for calibration.

## Results

### Influence of amino-terminal flanking residues on NifL activity

**Figure 2:**
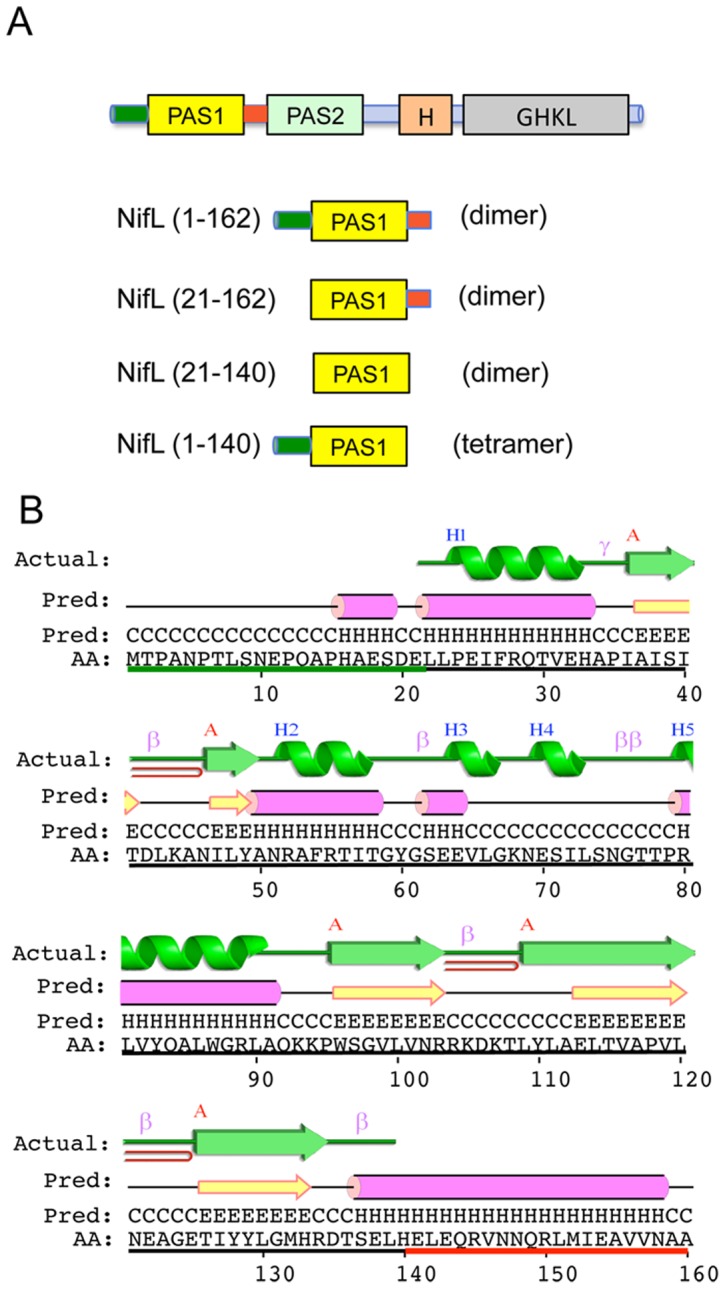
Structural features of the NifL PAS1 domain and its flanking regions. (A) Summary of NifL fragments used to analyse the influence of flanking regions on the properties of the PAS1 domain, indicated below the domain architecture of the complete protein. The oligomerisation state of each fragment, as determined by size exclusion chromatography in this work, is indicated in brackets. (B) The predicted secondary structure of NifL residues 1–160 as determined by the PSIPRED server [18] is shown above the amino acid sequence. Amino acids resolved in the crystal structure of the oxidized form of PAS1 [13] are underlined in black. The secondary structure plot for PDB code 2gj3: chain A (from PDBsum [30]) is displayed above the predicted structure. N-terminal and C-terminal flanking sequences are underlined in green and red respectively.

**Figure 3:**
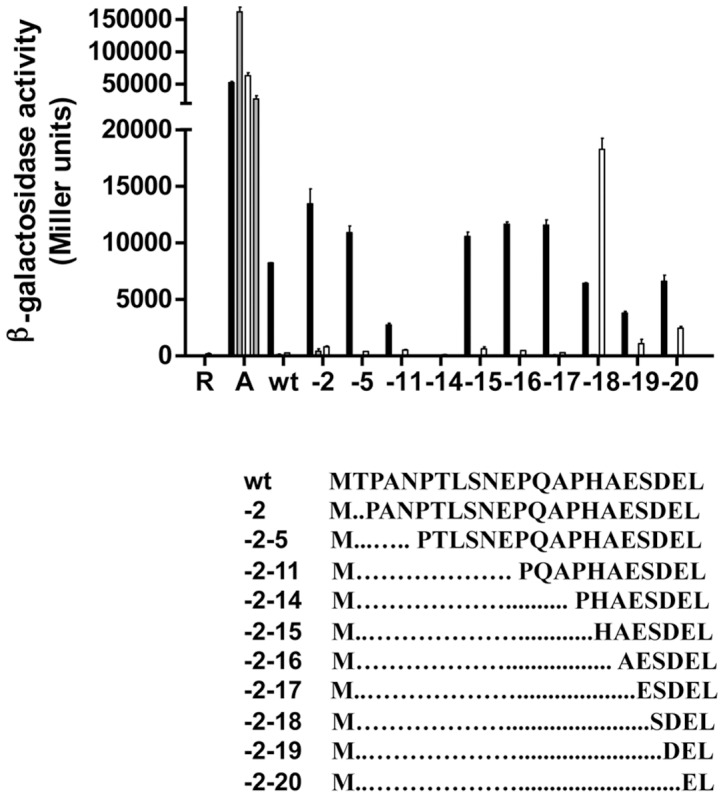
Influence of N-terminal truncations on the ability of NifL to regulate transcriptional activation by NifA. The number of residues missing from the N-terminus (including the initial methionine) is indicated numerically on the x axis and the sequence of each truncation is represented below. “R" indicates a control in which only the reporter plasmid was present. In all other cases, *nifA* was present on the plasmid together with *nifL* or its variants. “A" indicates the plasmid encodes an in-frame deletion, containing 65 C-terminal residues of NifL (pPR39). This plasmid acts as a control for NifA activity in the absence of active NifL [14]. “wt" indicates native NifL. Cultures were assayed for β-galactosidase activity as a reporter of NifA-mediated transcriptional activation from the *nifH_p_-lacZ* fusion on plasmid pRT22 as described previously [12]. Cultures were grown under the following conditions; anaerobically under nitrogen limitation with casein hydrolysate as the sole nitrogen source (black bars), anaerobically with (NH_4_)_2_SO_4_ as nitrogen source (grey bars), and aerobically with casein hydrolysate as sole nitrogen source (white bars). (Assays were also performed on cultures grown anaerobically in the presence of (NH_4_)_2_SO_4_, but β-galactosidase activities were too low to display on this scale.) All experiments were performed at least in duplicate with error bars denoting the standard error of the mean.

The crystal structure of the oxidised PAS1 domain of NifL from *A. vinelandii* is derived from an expression construct that includes residues 1–140. However, the 21 amino-terminal residues preceding the core PAS fold are unresolved in the crystal structure, presumably as a consequence of either proteolysis or disorder [13]. The N-terminus of NifL is relatively proline rich, but the PSIPRED server [18] predicts that residues 15–19 adopt an α-helical confirmation (Figure 2). We sought to investigate the influence of N-terminal residues on NifL function by expressing a range of truncated variants lacking some or all of the contributing residues. In all cases the amino terminal methionine was retained in these constructions and for *in vivo* experiments, protein expression was driven from the native *nifL* ribosome-binding site [14]. The sequences of these truncations are indicated in Figure 3. Truncated variants were examined for their ability to inhibit NifA activity in response to oxygen and fixed nitrogen. This was assessed using a two-plasmid system in *Escherichia coli* consisting of a reporter plasmid carrying a *nifH_p_-lacZ* fusion and a second plasmid from which *nifL* and *nifA* are constitutively co- expressed [14]. *A. vinelandii nifL* and *nifA* are co-transcribed and the native operon expresses stoichiometric levels of both proteins. As demonstrated previously, when wild-type NifL is present, NifA is only activated when oxygen and fixed nitrogen are limiting, under conditions appropriate for nitrogen fixation (Figure 3, black bars). In contrast, NifA is inhibited by NifL when either excess oxygen (open bars) or fixed nitrogen (grey bars) is present. Control experiments with a construct lacking most of the *nifL* coding sequence (an in-frame deletion containing 65 C-terminal residues of NifL) gave rise to higher levels of NifA activity than when wild-type was present (bars marked “A" in Figure 3). As noted previously, this suggests that NifL retains some inhibitory activity even under reducing, nitrogen-limiting conditions when nitrogen fixation is favoured [15], [16], [19].

Removal of residues 2–5 had no measurable effect on NifL activity but deletion of residues 2–11 increased the inhibitory activity of NifL in the absence of oxygen and fixed nitrogen (Figure 3A, black bars). Deletion of residues 2–14 resulted in complete repression of NifA activity under all conditions. Therefore, this truncation generates a ‘signal on’ state of NifL whereby the protein is apparently locked in a conformation that is inhibitory to NifA independently of environmental cues. Interestingly, residues 1–14 lie immediately upstream of the predicted α-helix formed by residues 15–19 (Figure 2B). However, a truncation lacking residues 2–15 had a wild-type phenotype and this was also the case with truncations lacking residues 2–17. A different phenotype was observed with truncations extending into residues 18 to 20. In this case the ability of NifL to inhibit NifA in the presence of oxygen was significantly diminished (Figure 3, compare black and white bars). We have previously observed that substitution of residues for alanine within the N-terminal amphipathic helix of PAS1 (denoted the A′α-helix or Ncap, comprising residues 22–34) results in defects in the ability of NifL to inhibit NifA in response to oxygen resulting in the so-called redox phenotype. We have argued that the redox phenotype generated by these mutants is a consequence of corrupting the integrity of the interfacial zipper-like motif formed by the A′α-helices in the oxidised PAS1 dimer [10]. Therefore the redox phenotype shown by the deletions extending through residues 18 to 20 may reflect increased disruption to this interface. With the exception of the 2–18 deletion, Western blotting analysis indicated that all the variants were as stable as wild-type NifL in all four growth conditions used for the ß-galactosidase assays (data not shown). However, the 2–18 deletion appeared to be less stable under oxygen replete, nitrogen-limiting conditions, which may explain why NifA activity was higher in this variant in the presence of oxygen compared with reducing conditions. Although some truncations clearly influence the response to oxygen, the fixed nitrogen signal, which is conveyed by the interaction of PII signal transduction proteins with the C-terminal GHKL domain of NifL [19], [20], [21] was unaffected by removal of N-terminal residues (Figure 2, grey bars).

To assess the importance of particular amino acids at the N-terminus, alanine scanning of individual residues spanning positions 12–18 inclusive was performed, with native alanine residues being substituted by glutamate. However, these substitutions were found to have little or no effect on the NifL activity with each mutant protein retaining a wild-type phenotype (Figure S1). This would support the notion that the secondary structure of the N-terminal region of NifL, rather than the property of specific residues, exerts an influence on protein activity.

### Analysis of the α helical linker connecting the PAS1 and PAS2 domains

Bioinformatic analysis of linker sequences connecting tandem PAS domains indicates that these linkers are likely to adopt an α-helical structure. They predominantly display an amphipathic sequence signature with heptad periodicity of hydrophobic residues, indicative of the formation of helical bundles or coiled-coils in multimeric proteins [6], [7]. The region of NifL between residues 139 and 159 is predicted to be α-helical (Figure 2B) and the COILS server (http://www.ch.embnet.org) predicts that this sequence may form a coiled-coil in the NifL dimer. Therefore the length and conformation of the linker between the PAS1 and PAS2 domains is likely to define their relative orientation and thus influence signal transmission.

**Figure 4:**
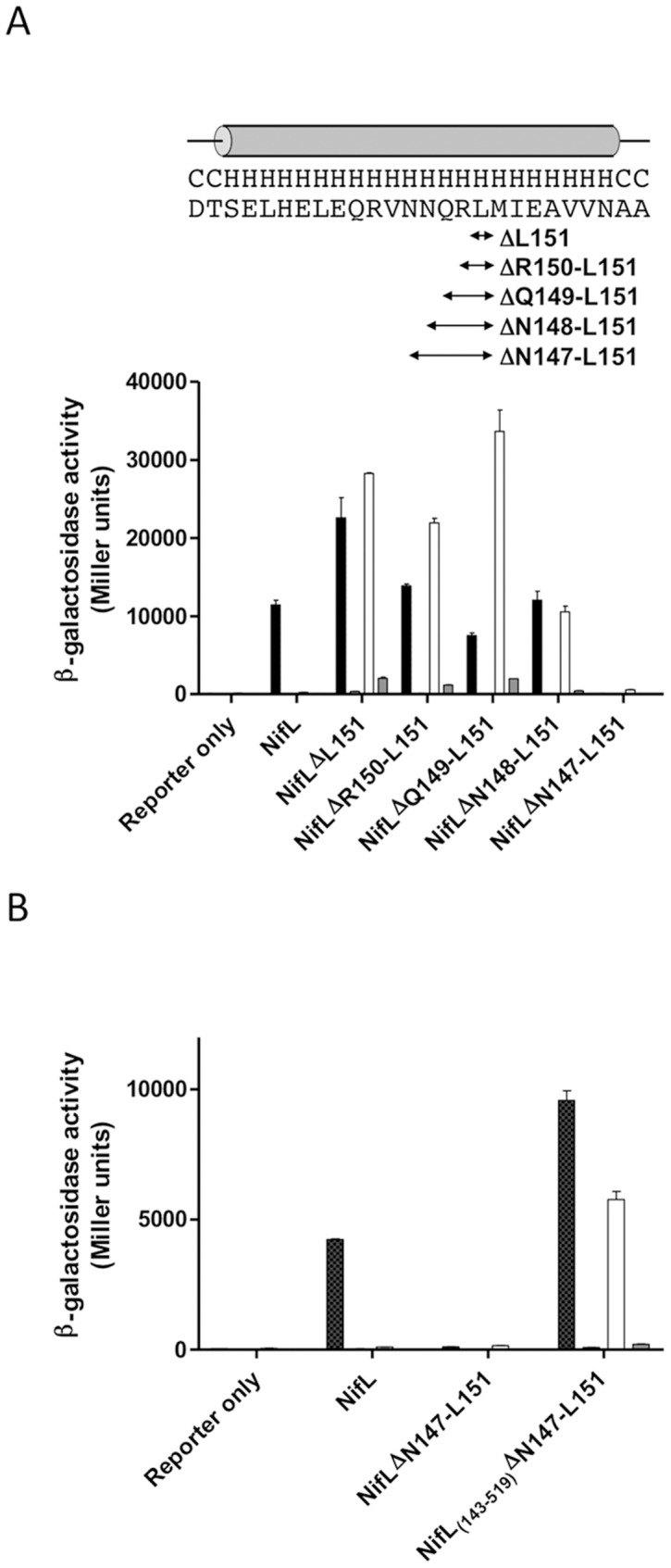
Influence of deletions in the α-helical linker connecting PAS1 and PAS2 on NifL activity. (A) Effect of linker deletions (indicated on the x axis) on the ability of NifL to control NifA activity (y axis). The sequence of the linker and the extent of the deletions are indicated above the graph. (B) Requirement for presence of the PAS1 domain for the “locked on" phenotype exhibited by the ΔN147-L151 deletion. Cultures were assayed for β-galactosidase activity as a reporter of NifA-mediated transcriptional activation from the *nifH_p_-lacZ* fusion on plasmid pRT22 as described previously [12]. Cultures were grown under the following conditions; anaerobically under nitrogen limitation with casein hydrolysate as the sole nitrogen source (black bars), aerobically with casein hydrolysate as sole nitrogen source (white bars) and aerobically with (NH_4_)_2_SO_4_ as nitrogen source (grey bars). (Cultures were also grown anaerobically in the presence of (NH_4_)_2_SO_4_, but activities were too low to display on this scale).

Alanine scanning of the predicted helical region between residues 139 and 150 revealed that seven out of the ten alanine substitutions failed to inhibit NifA activity in response to oxygen suggesting that this linker plays a crucial role in redox signal transduction (Figure S2). To further investigate the importance of this helical linker, five variant NifL proteins containing successive deletions between residues 148–151 were constructed. The influence of these deletions on the ability of NifL to inhibit NifA activity in response to oxygen and fixed nitrogen was analysed *in vivo* (Figure 4A). Western analysis indicated that all NifL proteins were stable under the four assay conditions tested (data not shown). Four of the deletions (NifLΔL151, NifLΔR150-L151, NifLΔQ149-L151 and NifLΔN148-L151) gave rise to a redox signalling phenotype whereby the NifL variants failed to inhibit NifA activity in the presence of excess oxygen but responded normally to fixed nitrogen. In contrast, the NifLΔN147-L151 variant inhibited NifA activity under all four conditions even when oxygen and fixed nitrogen were limiting (Figure 4A). Hence deletion of residues N147-L151 appears to lock NifL in a ‘signal-on’ conformation.

Previously we observed that ‘locked-on’ variants in either the PAS2 or the H domains of NifL did not require the presence of PAS1 to exert their inhibitory activity against NifA [12], [22]. To assess the requirement for PAS1 for the “locked-on" phenotype of the N147-L151 variant, the first 142 residues of NifL were removed to provide a truncated form of this variant lacking the first PAS domain. The ability of NifL_(143–519)_ΔN147-L151 to inhibit NifA-mediated transcriptional activation relative to the same variant possessing PAS1 was analysed *in vivo*. In contrast to the “locked-on" phenotype of the N147-L151 variant containing PAS1, the truncated variant retained the response to fixed nitrogen but was unable to inhibit NifA activity in response to oxygen (Figure 4B). Thus the NifL_(143–519)_ΔN147-L151 protein exhibited a redox signalling phenotype indicating that the PAS1 domain is required for the “locked on" phenotype generated by the deletion in the linker helix. This demonstrates that unlike ‘signal-on’ mutants previously isolated in PAS2 or in other downstream regions of the protein, perturbation of the linker helix reveals signal relay effects that require the N-terminal PAS1 domain.

### Influence of flanking residues on PAS1 oligomerisation

**Figure 5:**
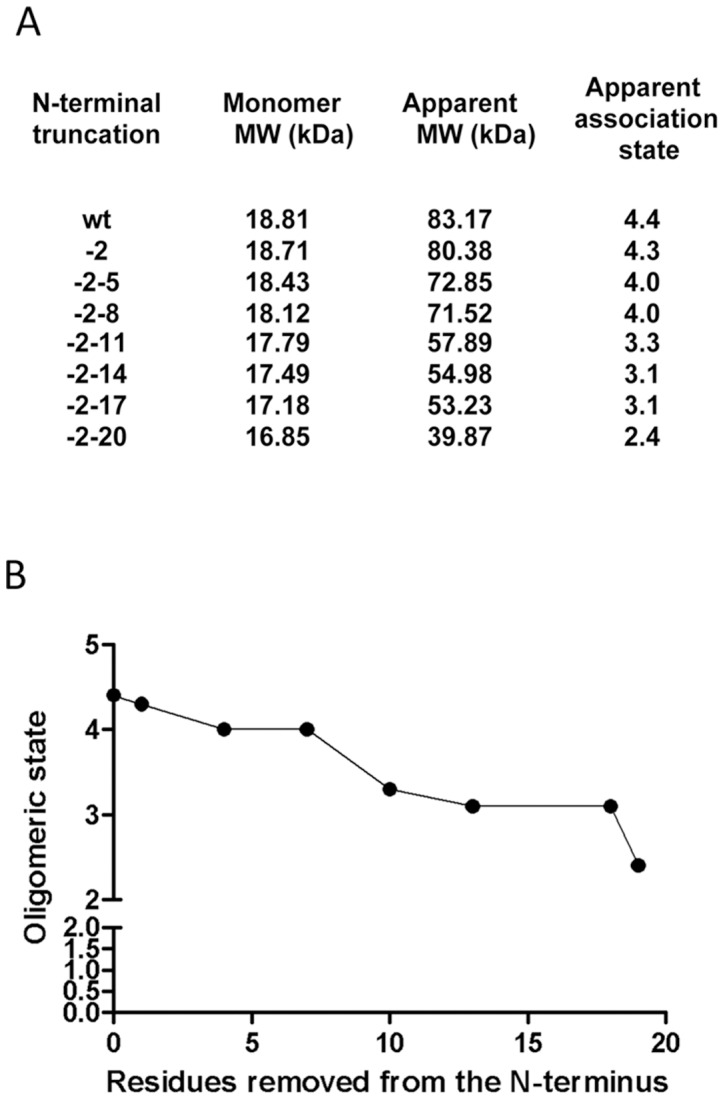
Influence of N-terminal residues on PAS1 oligomerisation state in the context of the 1–140 construct. (A) SEC analysis of wild-type and truncated variants (see Figure 3 for N-terminal sequences). In each case, the hexa-histidine-containing tag encoded by the pETNdeM-11 vector provides 26 additional residues at the N-terminus. SEC was performed at a protein concentration of 106 µM (expressed in monomeric terms). (B) The association state determined in (A) for each protein is plotted against the number of residues removed from the N-terminus.

Previous studies of PAS domains have demonstrated that the oligomerisation state of the PAS module can have an important role in signal transduction [12], [23], [24], [25]. To assess the influence of the flanking regions on the oligomerisation state of the isolated PAS1 domain, we subjected constructs to size exclusion chromatography (SEC). As observed previously [10], [26], the fragment comprising residues 1–140 (Figure 2A), which lacks the C-terminal linker helix, eluted as a multimeric species with a molecular weight closest to that of a tetramer (Figure 5A). This association state represents an uncommon stoichiometry for PAS domains that are typically dimeric. Previous work in this laboratory using analytical ultracentrifugation has demonstrated that a protein construct comprising residues 1–284 (containing both the PAS1 and PAS2 domains) is dimeric in solution in contrast to the 1–140 PAS1 domain construct, which exhibits a strong tendency to form tetramers at protein concentrations ranging from 10–100 µM ([10] and data not shown). Taken together, this suggests that the tetrameric state of the isolated PAS1 protein comprising residues 1–140 may not represent a physiologically relevant state. To investigate the role of the N-terminal residues in oligomerisation we purified truncated derivatives of the 1–140 PAS1 construct and analysed their SEC elution profiles at an equivalent protein concentration (106 µM). Removal of up to 8 N-terminal residues had little influence on the association state, but truncations removing residues 11–17 resulted in elution as an apparent trimeric species. In contrast, the construct lacking the entire N-terminal extension (residues 21–140) eluted as a dimer (Figure 5). The inability of this construct to tetramerise may relate to the redox signalling phenotype of the −18 to −20 truncations (Figure 3), which we ascribe to disruption of the Ncap A′α helix. Successive removal of N-terminal residues therefore decreases the tendency of the PAS1 domain to form tetramers, suggesting that the first 20 residues of NifL have an important influence on the oligomerisation state of the PAS1 domain.

Surprisingly however, a PAS1 construct containing both the N-terminal region and the C-terminal linker helix (residues 1–162, Figure 2) eluted at a molecular weight corresponding to 2.5 monomers (Table S1). This suggests that the C-terminal linker helix exerts an influence on the N-terminal residues upstream of the core PAS fold in such a way as to disfavour PAS1 tetramerisation. Furthermore, with this longer construct, progressive removal of residues from the N-terminal flanking region had a relatively minor influence on the oligomerisation state, with PAS1 eluting as a wholly dimeric population upon removal of the first 20 residues (Table S1). This may infer that communication exists between the flanking regions of PAS1 and that the linker helix plays a role in controlling the oligomerisation state of the domain.

**Figure 6:**
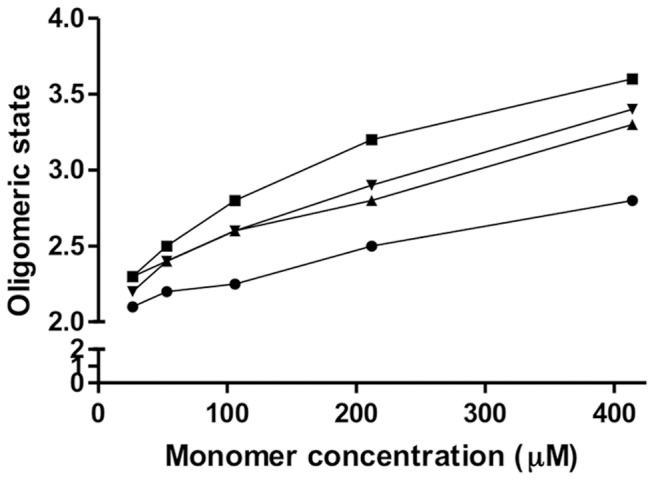
Influence of deletions in the α- helical linker on the oligomerisation state of the PAS1 domain in the 1–162 construct. The concentration dependence of the association state as assessed by SEC is plotted for: NifL_(1–162)_ (circles); NifL_(1–162)_ ΔL151 (upward triangles); NifL_(1–162)_ΔR150-L151 (downward triangles) and NifL_(1–162)_ΔN147-L151 (squares).

We reasoned that disruption of the C-terminal linker helix might break the apparent communication between this helix and other regions of the PAS1 domain, leading to a shift in oligomerisation state towards a tetrameric species. To assess the influence of residues in the linker helix on association state, we prepared constructs terminating at residue 162 for SEC analysis. In addition to the N147-L151 deletion, two deletions that had given rise to a redox phenotype (ΔL151 and ΔR150-L151) were also investigated. The oligomerisation state of each construct was assessed at five different protein concentrations (Figure 6). The construct with the native linker helix (residues 1–162) tended towards a dimeric state at low protein concentrations and eluted at an apparent molecular weight corresponding to 2.8 monomers at the highest injected concentration (424 µM) (Figure 6, circles). In contrast, all three deletion variants displayed an apparent shift in the dimer-tetramer equilibrium, eluting at higher molecular weights than the wild-type construct at all concentrations tested. The greatest shift towards the tetrameric state was exhibited by the construct lacking residues N147-L151 that gives rise to the ‘signal-on’ state of NifL. This deletion variant (NifL_(1–162)_ΔN147-L151) eluted at an apparent molecular weight corresponding to 3.6 monomers at the highest protein concentration tested (424 µM) (Figure 6, squares). This behaviour therefore supports a model in which the C-terminal linker helix plays a role in maintaining the PAS1 domain in the dimeric state and counteracts the ability of the N-terminal flanking residues to promote formation of the tetramer.

## Discussion

The data presented here demonstrate that both N- and C-terminal sequences flanking the core PAS1 domain of NifL influence signal transduction. Although the first 17 residues of the N-terminus are not apparently required for biological function and the properties of their amino acid side chains do not appear to be important for the response to the redox status, the conformation of the N-terminus seems to influence the signalling state. Removal of residues 2–14 generates a “locked on" phenotype, possibly as a consequence of rearrangement of a putative α-helix that extends from position 15–19. Truncations extending through this helix gave rise to defects in redox signalling, perhaps reflecting disruption to the A′α-helix (or Ncap) of the PAS1 domain, which we have shown previously to be important for redox signal transduction [10]. The failure of the −20 construct to convey the redox signal is particularly noteworthy as this represents the extent of the N-terminus resolved in the crystal structure. Therefore although the flavin in the crystal is in the oxidised form, there remains a possibility that the structure is not fully representative of the “on" (inhibitory) state. As summarized in Figure 2A our findings also suggest that the N-terminal flanking region is required for the unusual tetrameric association state of the isolated PAS1 domain, as its removal results in constructs that are predominantly dimeric in solution. Although, it is possible that molecular weight differences derived from size exclusion chromatography could be confounded by shape and folding affects in variant proteins, we have previously used a shape-independent method to analyse the association state of the PAS1 domain. Using equilibrium analytical ultracentifugation we established that, in contrast to a construct containing both PAS1 and PAS2, which like the native protein is dimeric in solution, the NifL(1–140) construct has a strong tendency to form a tetrameric species with a dissociation constant of 150 nM for the tetramer-dimer equilibrium [10]. Taken together with the evidence presented here, this strongly implies that the N-terminal flanking region drives the equilibrium towards the tetrameric state. This may explain why the isolated PAS1 domain is dimeric in the crystal structure as the initial 21 amino acids were not resolved [13]. Analogous effects of N-terminal residues on association state have been observed with other PAS domains. In these cases, alterations in quaternary structure can be attributed to changes in the conformation of the Ncap (A′α) helix, facilitated by the mobility of the N-terminal sequences [27], [28]. Overall our results suggest that the N-terminal region can influence the signal state, but the precise role of this sequence in signal transduction remains to be determined.

**Figure 7:**
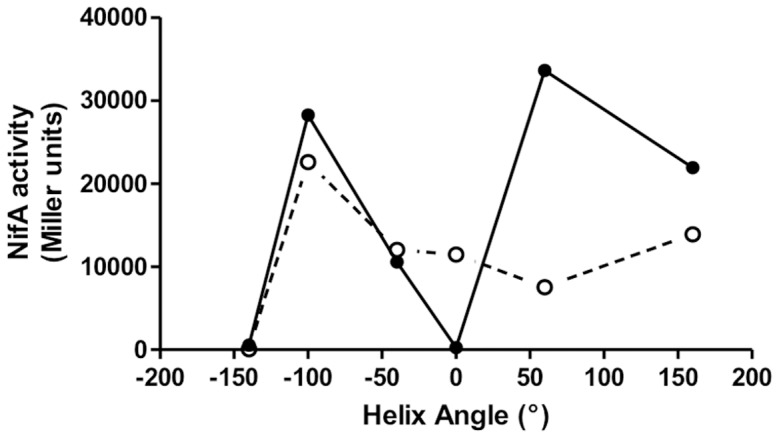
Influence of deletions in the α-helical linker on the ability of NifL to inhibit NifA plotted as the change in helix angle. The data is derived from the results in Figure 4A. Cultures were grown with casein hydrolysate as the sole nitrogen source, either under aerobic (solid circles, solid line) or anaerobic conditions (open circles, dotted line).

Many PAS domains contain flanking C-terminal amphipathic α-helices (sometimes referred to as the J α-helix) that either connect sensory domains with effectors or link together multiple sensory modules. Linkers connecting tandem PAS domains are predicted to adopt an α-helical conformation and the integrity of linker has been demonstrated to be important for signal transduction [7]. In the case of the NifL inter-domain linker, alanine scanning mutagenesis yielded numerous “redox signalling" variants. Moreover, deletion of residues in the linker resulted in severe effects on signal transmission, as might be expected if these deletions significantly alter the relative orientation of the PAS1 and PAS2 domains. When NifL activity (measured as inhibition of NifA activity) is plotted against the change in helix angle (Figure 7), the deletion of five residues gives rise to a change in helix angle of −140°, which results in a form of NifL that adopts an inhibitory conformation irrespective of the redox state of the PAS1 domain (i.e. NifA activity is very low either in the presence or absence of oxygen). At helix angles between −100° and −50° the redox status had little influence on NifL activity. Increases in the helix angle resulted in higher levels of activity under oxidising compared with reducing conditions, although we cannot rule out the possibility that this is a consequence of small changes in protein stability. In the presence of oxygen there appears to be periodicity in the relationship between the helix angle and NifL activity (Figure 7). These helical effects on signal transduction relate to interdomain signal relay rather than to downstream conformational changes, since we found that the constitutive “on state" signal exhibited by the five residue deletion, reverts to the “off state" when the PAS1 domain is removed. Taken together, these results indicate that the phasing of the PAS1-PAS2 linker helix is important for redox signal relay and suggests a model whereby signals are transmitted along a coiled-coil linker in the form of torque or helical rotation. This mechanism has been proposed for several PAS-containing proteins [6], [29]. In a recent study that combined experiments on chimeric PAS sensor proteins with the available structural data on tandem PAS domains, it was observed that multiple PAS domains are commonly arranged along a linear axis such that the N-terminus of the second domain is adjacent to the C-terminus of the first (i.e. they are oriented “head-to-tail"). The authors postulate that addition/reduction of torques along this axis provides a means of integrated signal output from multiple sensory PAS domains [7]. It has also been proposed that the C-terminal Jα helices protruding from the sensory PAS domains of the YtvA and FixL proteins relay signals to effector domains via a similar mechanism [6], [29].

Our findings have also uncovered a role for the linker helix in controlling the oligomerisation state of PAS1 by preventing tetramerisation of the core PAS domain (summarised in Figure 2A). This supports the notion that the linker forms an α-helical coiled-coil, which is likely to be disrupted by the deletions and hence the tendency of the linker to maintain PAS1 in the dimeric state is lost in the linker deletion variants. The opposing activities of the N-terminal sequence (promotion of the tetrameric state) and the C-terminal linker helix (maintenance of the dimeric state) may serve to maintain the tandem PAS domains in a parallel configuration along a central helical spine, as has been observed in multiple PAS domain proteins [7]. Communication between the N-terminal and C-terminal flanking sequences of PAS1 may serve to couple signal perception to signal transmission, resulting in transient dissociation of the PAS2 dimer [12] as has been observed in other sensory proteins with multiple PAS domains [23], [25]. Overall our results support an emerging picture in which helical regions outside the core PAS fold serve to propagate and potentially amplify signals between distal and proximal sensory domains.

## Supporting Information

Figure S1 ***In vivo*** **activity of alanine or glutamate substitutions in the N-terminal region of NifL.** Cultures were grown under the following conditions; anaerobically under nitrogen limitation (de-repressing conditions) with casein hydrolysate as the sole nitrogen source (green bars), aerobically with casein hydrolysate as sole nitrogen source (yellow bars) and aerobically with (NH_4_)_2_SO_4_ as nitrogen source (red bars). (Cultures were also grown anaerobically with (NH_4_)_2_SO_4_ as nitrogen source, but β-galactosidase activities in this case were too low to be visible on this scale.) Cultures were assayed for β-galactosidase activity as a reporter of NifA-mediated transcriptional activation from the *nifH_p_-lacZ* fusion on plasmid pRT22 as described previously [12]. All experiments were performed at least in duplicate with error bars denoting the standard error of the mean.
(TIF)

Figure S2 **Influence of alanine substitutions in the α-helical linker on the ability of NifL to inhibit NifA-mediated transcriptional activation from a** ***nifH-lacZ*** **reporter fusion** ***in vivo*** **.** Cultures were grown under the following conditions; anaerobically under nitrogen limitation (de-repressing conditions) with casein hydrolysate as the sole nitrogen source (green bars), aerobically with casein hydrolysate as sole nitrogen source (yellow bars) and aerobically with (NH_4_)_2_SO_4_ as nitrogen source (red bars). (Cultures were also grown anaerobically with (NH_4_)_2_SO_4_ as nitrogen source, but β-galactosidase activities in this case were too low to be visible on this scale.) Cultures were assayed for β-galactosidase activity as a reporter of NifA-mediated transcriptional activation from the *nifH_p_-lacZ* fusion on plasmid pRT22 as described previously [12]. All experiments were performed at least in duplicate with error bars denoting the standard error of the mean.
(TIF)

Table S1 **Influence of amino-terminal flanking residues on oligomerisation state in the 1–162 PAS1 construct of NifL.**
(DOCX)
