# Supplementary figures and images for: Influence of PAS Domain Flanking Regions on Oligomerisation and Redox Signalling By NifL
Source: PLoS One. 2012 Oct 8;7(10):e46651. doi: 10.1371/journal.pone.0046651 (PMC3466315; doi:10.1371/journal.pone.0046651)

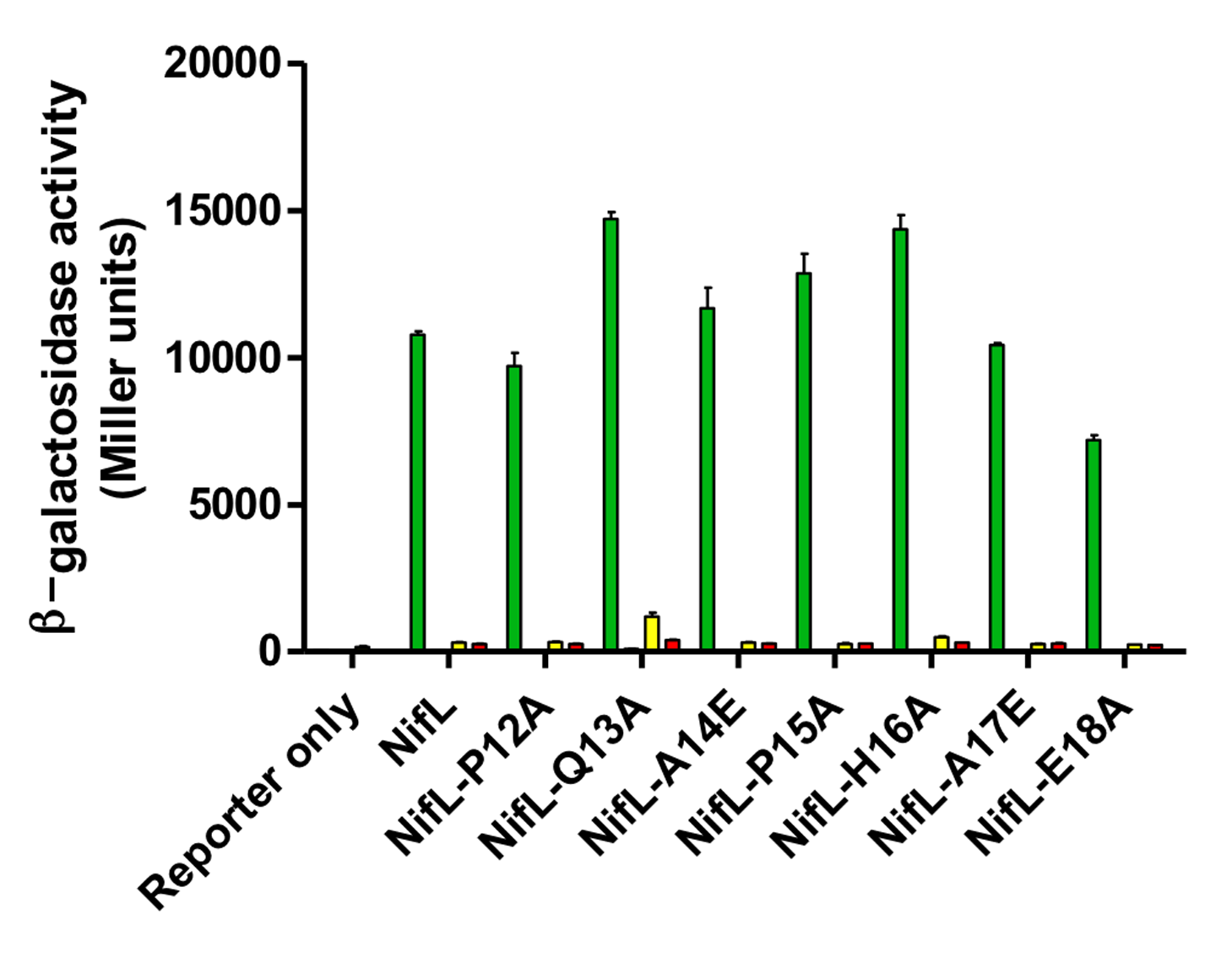

Supplement: Figure S1 — In vivo activity of alanine or glutamate substitutions in the N-terminal region of NifL. Cultures were grown under the following conditions; anaerobically under nitrogen limitation (de-repressing conditions) with casein hydrolysate as the sole nitrogen source (green bars), aerobically with casein hydrolysate as sole nitrogen source (yellow bars) and aerobically with (NH4)2SO4 as nitrogen source (red bars). (Cultures were also grown anaerobically with (NH4)2SO4 as nitrogen source, but β-galactosidase activities in this case were too low to be visible on this scale.) Cultures were assayed for β-galactosidase activity as a reporter of NifA-mediated transcriptional activation from the nifHp-lacZ fusion on plasmid pRT22 as described previously [12]. All experiments were performed at least in duplicate with error bars denoting the standard error of the mean. (TIF) [file pone.0046651.s001.tif]

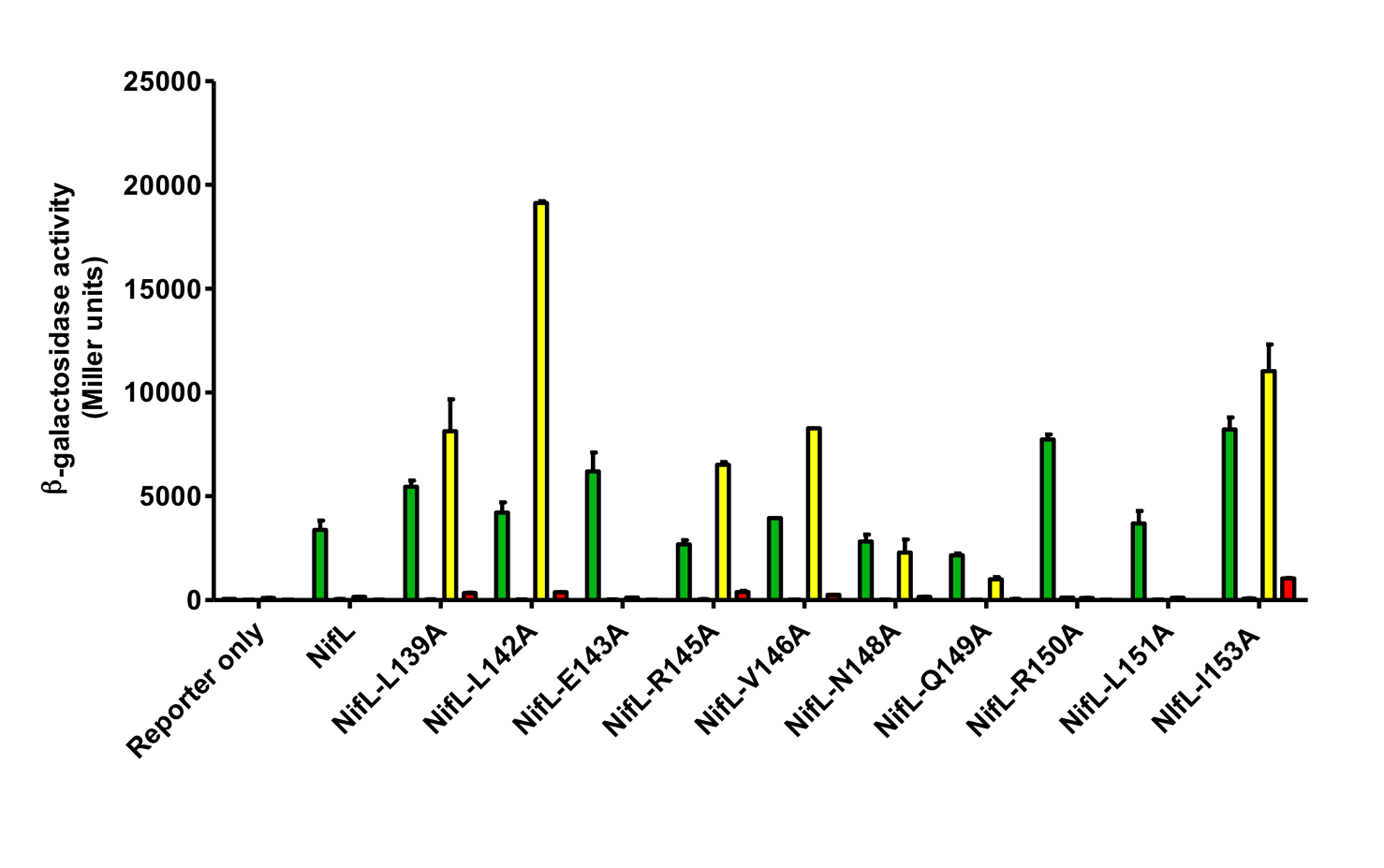

Supplement: Figure S2 — Influence of alanine substitutions in the α-helical linker on the ability of NifL to inhibit NifA-mediated transcriptional activation from a nifH-lacZ reporter fusion in vivo . Cultures were grown under the following conditions; anaerobically under nitrogen limitation (de-repressing conditions) with casein hydrolysate as the sole nitrogen source (green bars), aerobically with casein hydrolysate as sole nitrogen source (yellow bars) and aerobically with (NH4)2SO4 as nitrogen source (red bars). (Cultures were also grown anaerobically with (NH4)2SO4 as nitrogen source, but β-galactosidase activities in this case were too low to be visible on this scale.) Cultures were assayed for β-galactosidase activity as a reporter of NifA-mediated transcriptional activation from the nifHp-lacZ fusion on plasmid pRT22 as described previously [12]. All experiments were performed at least in duplicate with error bars denoting the standard error of the mean. (TIF) [file pone.0046651.s002.tif]
