# Supplementary material for: Influence of PAS Domain Flanking Regions on Oligomerisation and Redox Signalling By NifL
Source: PLoS One. 2012 Oct 8;7(10):e46651. doi: 10.1371/journal.pone.0046651 (PMC3466315; doi:10.1371/journal.pone.0046651)
Supplement: Table S1 — Influence of amino-terminal flanking residues on oligomerisation state in the 1–162 PAS1 construct of NifL. (DOCX) [file pone.0046651.s003.docx]

**Table S1**

Monomeric Apparent

Expression molecular weight molecular weight Apparent

| pRL294 (1-162)^c^ 21.26 52.48 2.5  pRL295 (3-162) 21.16 52.14 2.5  pRL296 (9-162) 20.56 49.18 2.4  pRL297 (18-162) 19.63 45.12 2.3  pRL298 (21-162) 19.30 37.71 2.0 |
| --- |

construct^a^ (kDa) (kDa)^b^ oligomeric state

^a^ The hexa-histidine-containing tag encoded by the pETNdeM-11 vector provides 26 additional residues at the N-terminus.

^b^ SEC was performed at a protein concentration of 106 μM (expressed in monomeric terms).

^c^ Plasmid pRL294 encoding NifL_(1-162)_ was prepared by amplifying the *nifL* gene from pPR34 with the forward primer T7 (5’- TAATACGACTCACTATAGGG-3’) and the reverse primer NifL162Rev (5’CGAAGGATCCTCACGCCGGGGCGGCGTTGACCAC-3’) that introduces a stop codon and a *Bam*HI restriction endonuclease site. The PCR products were purified (Qiagen PCR purification kit) and digested with the restriction endonucleases NdeI and BamHI (Fermentas FastDigest enzymes). The digested fragment was cloned into pETNdeM-11. The plasmids pRL295 – pRL298 encoding N-terminal truncations of NifL terminating at residue 162, were prepared as pRL294 with the exception that the forward primer was designed to anneal at the desired starting codon.
